# *Streptococcus pseudoporcinus*: Case Reports and Review of the Literature

**DOI:** 10.1155/2020/4135246

**Published:** 2020-04-22

**Authors:** Shanza Khan, Ting Ting Wong, Nishant Prasad, Benjamin Lee, Carl Urban, Sorana Segal-Maurer, Glenn Turett

**Affiliations:** ^1^Division of Infectious Diseases, NewYork Presbyterian Queens, Flushing, NY, USA; ^2^NewYork-Presbyterian Brooklyn Methodist Hospital, Brooklyn, NY, USA; ^3^Department of Cardiothoracic Surgery, NewYork Presbyterian Queens, Flushing, NY, USA

## Abstract

*Streptococcus pseudoporcinus* is a beta-hemolytic Gram-positive, catalase-negative, nonmotile coccus arranged in short chains, usually found in the female genitourinary tract and differentiated from *Streptococcus porcinus* in 2006. Only two human infections associated with this organism have been reported to date: one in a patient with a first digit wound infection and another with lower extremity cellulitis. We describe two novel cases of *Streptococcus pseudoporcinus* causing endocarditis in one and pneumonia with empyema in another, illustrating the potential of these bacteria to cause severe invasive and life-threatening disease.

## 1. Background

*Streptococcus pseudoporcinus* is a beta-hemolytic Gram-positive, catalase-negative, nonmotile coccus arranged in short chains and first described in 2006 [1]. Although it was phenotypically identified as *Streptococcus porcinus*, sequencing data differentiated it as a novel species [1]. Only two reported human infections implicated this bacterium including a wound infection of a finger as a result of trauma and another with left lower leg cellulitis associated with stasis dermatitis [2, 3]. We describe two additional cases, one causing endocarditis and a second with pneumonia and empyema. Both highlight the potential of this organism to cause invasive infections in environments outside of its normal habitat and illustrate that infections caused by *Streptococcus pseudoporcinus* can lead to severe and life-threatening disease.

## 2. Case 1

**Figure 1:**
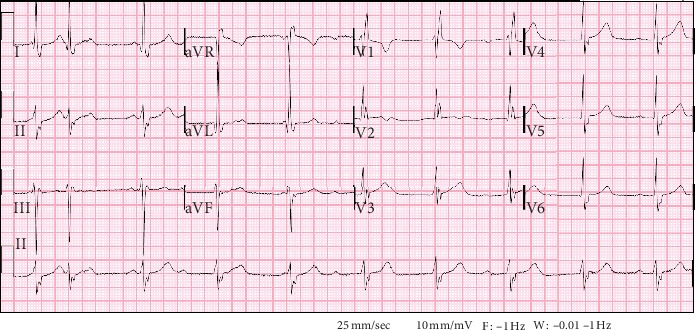
Admission ECG showing a third-degree heart block with a wide complex escape rhythm, right bundle branch block, left anterior fascicular block, and left ventricular hypertrophy.

**Figure 2:**
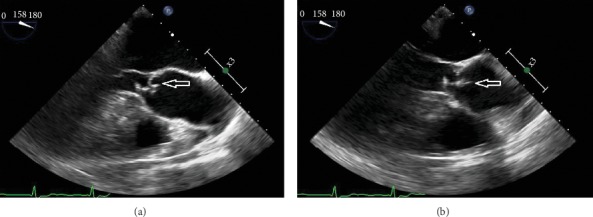
Aortic valve (a) with mobile echodensity (b) on the ventricular aspect of the right coronary cusp.

**Figure 3:**
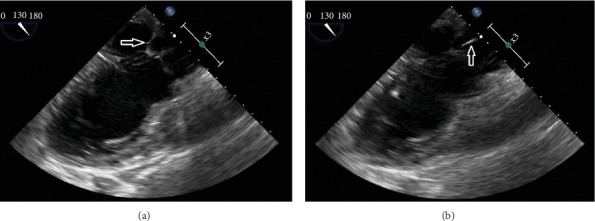
Mitral valve (a) with a mobile echodensity (b) on the atrial aspect of the valve.

An 81-year-old Colombian male presented to the hospital with a 4-day history of productive cough and 2-week history of fevers and chills. His past medical history was significant for hypertension, hyperlipidemia, diabetes mellitus, and chronic heart failure with preserved ejection fraction. In the Emergency Department (ED), he was febrile to 38.7°C and bradycardic at 55 beats/min. Initial physical exam demonstrated an obese, diaphoretic male with poor dentition. He was mildly tachypneic with decreased breath sounds and an audible grade III/VI systolic ejection murmur in the right second intercostal space. There was significant pitting edema in his extremities, with chronic venous stasis changes, and his right lower extremity was warm and erythematous. Laboratory studies revealed a leukocytosis of 25,000 cells/*μ*l (4.8–10.8 cells/*μ*l) with 87% neutrophils, 7% monocytes, 2% lymphocytes, and 4% band forms, and serum chemistries were remarkable for hyperkalemia with a potassium level of 6.5 mmol/L (3.5–5.5 mmol/L) and elevated creatinine of 1.67 mg/dL (0.7–1.3 mg/dL). An electrocardiogram revealed a third-degree heart block with a wide complex escape rhythm (Figure 1). He was admitted to the Cardiac Intensive Care Unit, and electrophysiology (EP) service was consulted for external transvenous pacer placement. An infectious disease consultation was obtained to evaluate optimal timing for placement of a permanent pacemaker as the initial blood cultures were reported to contain Gram-positive cocci in chains, later identified as *Streptococcus pseudoporcinus* by the clinical microbiology laboratory using the Vitek 2™ system and the VT2 GP ID™ card. Transthoracic echocardiogram demonstrated a preserved ejection fraction with moderate aortic regurgitation (AR) but without evidence of valvular vegetations. The infectious disease consultant considered the source of his bacteremia to be due to right lower extremity cellulitis. The patient received 2 g of intravenous ceftriaxone daily. Repeat blood cultures obtained on hospital day 3 remained negative after five days so permanent pacemaker placement was scheduled. Upon transfer to the EP lab, the patient experienced respiratory failure requiring mechanical ventilation. The chest radiograph demonstrated dense airspace disease of bilateral lung fields. He continued to be febrile despite appropriate parenteral therapy. A repeat fever workup was unrevealing other than the urinalysis demonstrating proteinuria and increased RBCs. His creatinine remained elevated throughout his stay. Fevers were postulated to be due to beta-lactam agent use; therefore, the antibiotic regimen was changed to intravenous vancomycin. Cardiothoracic surgery was consulted for the moderate AR seen on an initial echocardiogram, and they recommended a transesophageal echocardiogram (TEE). During the procedure, the patient suffered a pulseless electrical activity (PEA) cardiac arrest with return of spontaneous circulation after five minutes of cardiopulmonary resuscitation. Repeat chest radiograph demonstrated pulmonary edema. The TEE revealed a mobile echodensity seen on the ventricular aspect of the right coronary cusp of the aortic valve with severe AR and a mobile echodensity on the atrial aspect of the mitral valve with moderate mitral regurgitation (Figures 2 and 3). In spite of full critical care support, he again suffered PEA arrest and eventually the family decided to withdraw care and he died on hospital day 23.

## 3. Case 2

**Figure 4:**
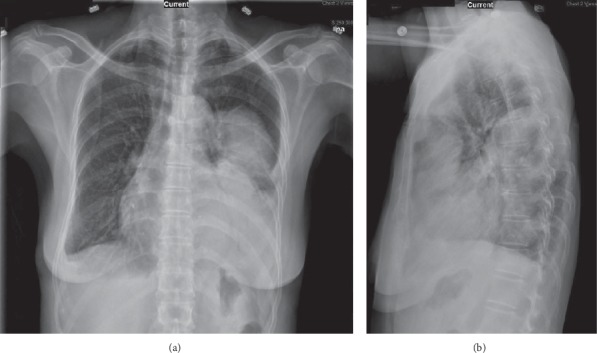
Chest X-ray images of the confluent opacity at the left lower hemithorax. Frontal view (a) and lateral view (b).

**Figure 5:**
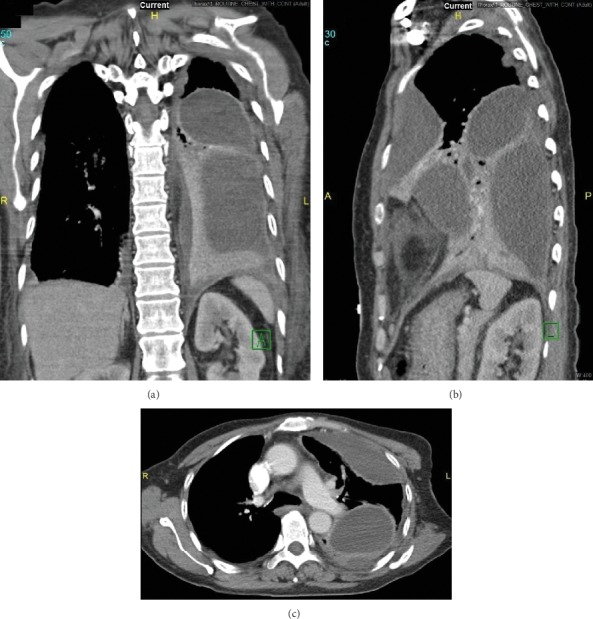
Chest CT images of the multiloculated empyema. Coronal view (a), sagittal view (b), and axial view (c).

**Figure 6:**
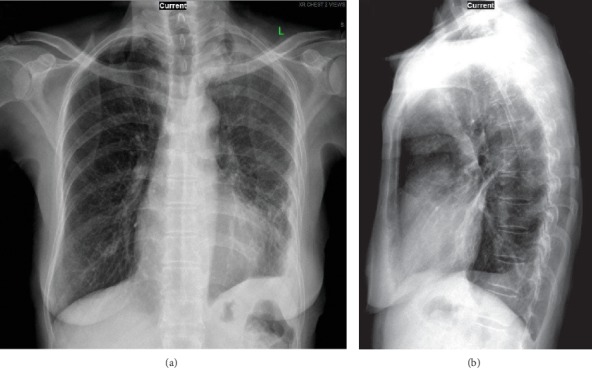
Chest X-ray images of the improvement of the opacity at the left lower hemithorax at week 3 of intravenous ertapenem therapy. Frontal view (a) and lateral view (b).

A 72-year-old Chinese female presented to the ED with productive cough, chest pain, fevers, and chills for one week. In the ED, the patient was found to have a fever of 38.3°C and an oxygen saturation of 93% on room air. Initial physical examination revealed a thin female with a dry cough, occasional crackles over the left middle lung field, and decreased breath sounds over the left lower lung field. Laboratory findings revealed a leukocytosis of 15,000 cells/*μ*l (4.8–10.8 cells/*μ*l), with 86% neutrophils, 5% lymphocytes, and 8% monocytes, and serum chemistries were remarkable for mild hyponatremia of 130 mmol/L (136–145 mmol/L) and a negative polymerase chain reaction (PCR) for the influenza virus. A chest radiograph demonstrated an opacity in the left lower lobe consistent with consolidation suggestive of pneumonia (Figure 4). She subsequently received empiric piperacillin-tazobactam 4.5 g IV every 8 hours and azithromycin 500 mg IV daily for presumed bacterial pneumonia. A computed tomogram of the chest performed the next day demonstrated a large multiloculated pleural effusion in the left lung with collapse of the left upper lobe (Figure 5). The patient underwent video-assisted thoracoscopic surgery and decortication of the left lung. Intraoperative findings included thickened and acutely inflamed pleura with purulent pleural fluid. Tissue specimens were sent to the microbiology laboratory for analysis. The Gram stain of both lung tissue and pleural fluid revealed few white blood cells, few Gram-positive cocci in pairs, and few Gram-negative rods. The Gram-positive organism was later identified by the clinical microbiology laboratory, using the Vitek 2^™^ system and the VT2 GP ID^™^ card, as *Streptococcus pseudoporcinus*. The isolate was sensitive to all antibiotics tested except tetracycline. Antibiotics were de-escalated to ceftriaxone 1 g IV daily. Several days later, a Gram-negative organism was identified by the same system as a beta-lactamase positive *Prevotella oris*. The patient was discharged to the outpatient parenteral antimicrobial therapy unit (OPAT) where her antibiotic regimen was changed to ertapenem 1 g IV daily to treat both organisms. The patient did well, and repeat chest radiograph after three weeks of intravenous therapy demonstrated improvement of the previously seen pleural and parenchymal opacity at the left lung base (Figure 6). The patient was subsequently discharged from the OPAT after completing four weeks of antibiotics.

## 4. Discussion

We present two novel cases of severe invasive infections due to *Streptococcus pseudoporcinus* with varying outcomes. Our first patient met modified Duke's criteria for definitive endocarditis, one of the two major criteria (endocardial involvement) and three minor criteria (fever, glomerulonephritis, and positive blood cultures with an organism not typically associated with endocarditis). Although *Streptococcus* species are commonly associated with endocarditis, this is the first known report of *Streptococcus pseudoporcinus* causing endocarditis.

Our second patient presented with empyema whose fluid and tissue specimens yielded *S. pseudoporcinus* and *Prevotella oris*. The definitive source of this patient's *S. pseudoporcinus* is unclear. As *Prevotella oris* can colonize the oropharynx, gastrointestinal tract, vagina, and genitourinary tract, we postulate that the *S. pseudoporcinus* may have originated in her oropharynx or gastrointestinal tract and ultimately led to her clinical disease via aspiration.

*Streptococcus pseudoporcinus* is a beta-hemolytic Gram-positive, catalase-negative, nonmotile coccus arranged in short chains and can sometimes be confused with *Streptococcus agalactiae* (group B *Streptococcus*) because of its recovery from vaginal-rectal specimens, similar colony morphology and biochemical reactions, and frequent cross-reactivity with Lancefield group B reagents [1, 4]. In our case, identification was made using the VT2 GP ID™ card which is able to differentiate *S. porcinus* from *S. pseudoporcinus*. It was first described in 2006 after several human isolates phenotypically identified as *Streptococcus porcinus* were sequenced and found to be over 2% dissimilar to any other *Streptococcus* species [1]. Reported human infections associated with this organism include a wound infection of the finger and lower extremity cellulitis [2, 3]. However, a recent publication described a patient where *S. pseudoporcinus*, initially identified in a vaginal-rectal culture as *Streptococcus porcinus* by the matrix-assisted laser desorption ionization-time of flight mass spectrometry system (Bruker Microflex, Biotyper software V.3.0, and database v.3.1.66), was subsequently confirmed as *S. pseudoporcinus* after DNA sequencing [5] Thus, many previous reports of *Streptococcus porcinus* causing infection may in fact have been due to *Streptococcus pseudoporcinus*, considering the difficulties in accurate identification.

S. pseudoporcinus isolates are usually susceptible to beta-lactams, erythromycin, clindamycin, vancomycin, and trimethoprim-sulfamethoxazole; most of them are resistant to tetracycline, as was observed in our cases [6]. However, one case report documented a multidrug-resistant *S. pseudoporcinus* isolate [3]. An IgG-degrading enzyme designated as IgdE was recently discovered that is highly specific for members of some of the *Streptococcus* species [7]. The *S. pseudoporcinus* IgdE protease appears to have specificity towards human IgG. The cleavage of the IgG subtypes might be a contributing factor for evading key immune defense mechanisms and progression to invasive disease [7].

## 5. Conclusion

*Streptococcus pseudoporcinus* is a relatively newly identified organism which is usually isolated from the female genitourinary tract. We presented two novel cases in whom this bacterium was implicated in both endocarditis and pneumonia with empyema, which highlights the potential of this organism to cause infection in environments outside of its identified habitat. Our two cases also illustrate that infections caused by *Streptococcus pseudoporcinus* can lead to severe and life-threatening disease.
